# Matrix first, minerals later: fine-tuned dietary phosphate increases bone formation in zebrafish

**DOI:** 10.1093/jbmrpl/ziae081

**Published:** 2024-06-18

**Authors:** Silvia Cotti, Claudia Di Biagio, Ann Huysseune, Wolfgang Koppe, Antonella Forlino, P Eckhard Witten

**Affiliations:** Biology Department, Evolutionary Developmental Biology, Ghent University, 9000 Gent, Belgium; Department of Molecular Medicine, Biochemistry Unit, University of Pavia, 27100 Pavia, Italy; Biology Department, Evolutionary Developmental Biology, Ghent University, 9000 Gent, Belgium; Department of Biology, University of Rome Tor Vergata, 00133 Rome, Italy; Biology Department, Evolutionary Developmental Biology, Ghent University, 9000 Gent, Belgium; Department of Zoology, Charles University, 12800 Prague, Czech Republic; SimplyFish AS, 4011 Stavanger, Norway; Department of Molecular Medicine, Biochemistry Unit, University of Pavia, 27100 Pavia, Italy; Biology Department, Evolutionary Developmental Biology, Ghent University, 9000 Gent, Belgium

**Keywords:** animal models, matrix mineralization, osteoblasts, collagen, analysis/quantitation of bone

## Abstract

Bone matrix formation and mineralization are two closely related, yet separated processes. Matrix formation occurs first, mineralization is a second step strictly dependent on the dietary intake of calcium and phosphorus (P). However, mineralization is commonly used as diagnostic parameter for bone-related diseases. In this context, bone loss, often characterized as a condition with reduced bone mineral density, represents a major burden for human health, for which increased dietary mineral intake is generally recommended. Using a counterintuitive approach, we use a low-P diet followed by a sufficient-P intake to increase bone volume. We show in zebrafish by histology, qPCR, micro-CT, and enzyme histochemistry that a two-months period of reduced dietary P intake stimulates extensive formation of new bone matrix, associated with the upregulation of key genes required for both bone matrix formation and mineralization. The return to a P-sufficient diet initiates the mineralization of the abundant matrix previously deposited, thus resulting in a striking increase of the mineralized bone volume as proven at the level of the vertebral column, including vertebral bodies and arches. In summary, bone matrix formation is first stimulated with a low-P diet, and its mineralization is later triggered by a sufficient-P dietary intake. In zebrafish, the uncoupling of bone formation and mineralization by alternating low and sufficient dietary P intake significantly increases the bone volume without causing skeletal malformations or ectopic mineralization. A modification of this approach to stimulate bone formation, optimized for mammalian models, can possibly open opportunities to support treatments in patients that suffer from low bone mass.

## Graphical Abstract

**Graphical Abstract:**
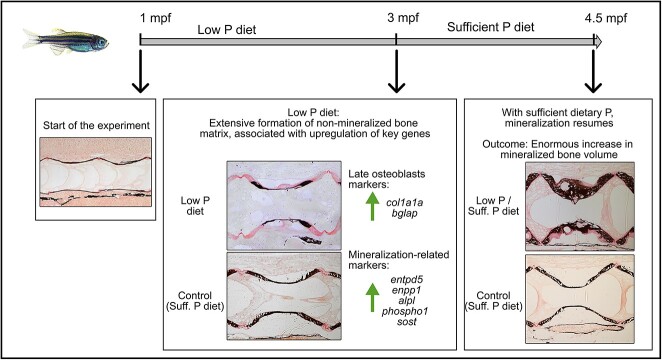

## Introduction

Studies on human bone show that mineralization is a biphasic process. The primary phase of mineralization (about 50%) is rapid, whereas completion of mineralization is slow and can last for months or years. Under healthy conditions, bone matrix formation and bone matrix mineralization, both in human and zebrafish, are tightly linked, with the former anticipating the latter. In humans, bone matrix formation precedes the start of mineralization by a maximum of 15 days,^1^ in zebrafish by 5 to 6 hours.^2^ Due to the connection of the two processes and the usually short time interval between them, bone formation is often equated with mineralization.^3^ There are also technical reasons for this equation. Compared to collagen secretion and organic matrix assembly, mineralization is easier to identify and to quantify. X-ray-based technologies such as DXA for humans and micro-computed tomography (micro-CT) for smaller animals allow to measure the mineral phase of bone to establish BMD and BMC, respectively, a diagnostic value for the amount of bone.^4^ Histological techniques measure bone growth and remodeling by labelling the mineralization front with fluorochromes such as tetracycline, alizarin, or calcein.^5^^,^^6^

Equating bone mineralization with bone formation is, however, not without pitfalls when it comes to bone quality assessment. For example, fracture resistance is not directly proportional to the amount of bone mineralization. Bone is a composite material, made from a collagenous matrix and minerals, two components with different properties. The collagenous matrix provides toughness (fracture-resistance) and the minerals increase the bones’ stiffness (bending-resistance).^7^ The mineral phase alone is brittle and fractures easily. Bone strength largely depends on the non-mineralized matrix, specifically on the orientation of collagen fibers that become arranged according to the direction of mechanical load. Indeed, collagenous bone matrix without minerals can be tougher than mineralized bone.^7^ Likewise, human individuals affected by OI have over-mineralized bones, characterized by reduced mineral density, that fracture easily, hence “brittle bone” disease.^8^ On the other hand, one of the toughest bones known are deer antlers, an example of fast-forming bone in adult mammals with low mineral content (about half of a human femur bone) that is extremely fracture-resistant.^7^

Age and disease-related bone loss, which leads to osteoporosis and other types of skeletal fragility, constitute a major health burden for humans.^9^ Osteoporotic conditions derive from an imbalance between bone formation and bone resorption, in favor of the latter.^10^ Antiresorptive drugs help to reduce bone resorption, but they do not stimulate bone formation nor do they restore the initial bone volume.^10^ Bone turnover is instead reduced, causing an extensive secondary mineralization of the remaining bone that loses its elasticity and becomes more fragile.^7^^,^^10^ Age-related over-mineralization contributes to this condition. Over-mineralization makes bone brittle and it reduces the surface layer of non-mineralized bone matrix, the osteoid, that protects bone from resorption.^7^^,^^10^^,^^11^ Thus, rather than increasing the BMC, which is a standard approach in bone loss therapy, it is tempting to speculate that the stimulation of bone matrix formation may enhance fracture resistance and restoration of the protective osteoid layer. Indeed, intermittent treatment with PTH derivatives and the recently Food and Drug Administration (FDA)-approved use of antibodies directed against sclerostin, an endogenous inhibitor of the anabolic “Wingless-related integration site” (WNT) pathway,^12^ are therapeutic approaches for low bone mass diseases aiming at stimulating bone matrix formation.^13^

Besides drugs, it is well known that multiple environmental factors, including a balanced nutrition, exercise, and avoidance of adverse substances (smoking, excessive alcohol, and other drugs) positively impact bone quality.^14^ Concerning nutrition, numerous macro- and micronutrients are important for bone development and maintenance.^14^ Among macronutrients, calcium and phosphorus (P) are the main inorganic components of bone and their levels are regulated to ensure optimal skeletal conditions.^15^^,^^16^ In the study of bone health and mechanisms underlying skeletal diseases, the zebrafish *Danio rerio* has become an important model organism thanks to conserved basic processes of skeletal formation across gnathostomes.^17^^,^^18^ Given that teleosts, like tetrapods, depend on dietary P, they represent good models to explore the role of dietary P for bone mineralization.^19^ It is known that excess dietary P intake can reduce bone formation in humans.^20^ Furthermore, it has been shown that excess dietary P intake in rats promotes bone hypermineralization, which leads to increased bone stiffness and ultimately to a reduction of bone strength (maximal load).^21^ Conversely, recent studies on zebrafish and other teleost fish (Atlantic salmon) have drawn the attention to the importance of reduced dietary P levels for bone formation and bone quality.^22–26^ We have previously showed in zebrafish and salmon that a decrease of dietary P intake by 50% reduces the mineralization of both endoskeletal bone elements (i.e. the vertebral bodies and arches) and dermal bones (i.e. the fin rays, scales and operculum), but results in increased formation of non-mineralized bone matrix.^22^^,^^24^^,^^26^ The reduced dietary P intake was shown to improve the biomechanical properties by enhancing toughness and reducing stiffness,^23^^,^^24^^,^^26^ leading to a decreased fracture risk.^7^^,^^23^^,^^24^^,^^26^

The current study aims at strengthening our previous findings on the zebrafish model. Here we show that by feeding the animals a phosphorus (P)-reduced diet, the formation of new bone matrix in the vertebral column is accompanied by the upregulation of genes related to bone matrix formation together with genes that regulate bone mineralization. Interestingly, the new bone remains non-mineralized, but it quickly mineralizes as soon as the animals return to a P-sufficient diet. The result is a marked increase of the mineralized bone volume. A similar dietary treatment, optimized on mammalian models properly timing the two P diets, could eventually pave the way for a new approach to support therapies for patients that suffer from low bone mass diseases.

## Materials and methods

### Zebrafish husbandry

WT AB zebrafish were obtained from the European Zebrafish Research Center or from the Zebrafish Facility Ghent. The reporter zebrafish line *osc*:GFP was a gift from Dr A. Willaert, Ghent University Hospital and was originally obtained from Dr S. Schulte-Merker,^27^ Institute for Cardiovascular Organogenesis and Regeneration. Zebrafish embryos were kept in fish water (1.2 mM NaHCO_3_, 0.01% instant ocean, 1.4 mM CaSO_4_, 0.0002% methylene blue) at 28°C until 7 days post-fertilization (dpf), then grown at 28°C, pH 7.5 on a 14/10 light/dark cycle. At the end of the experiment, fish were euthanized by tricaine (3-amino benzoic acidethylester) overdose (0.3%) and fixed for further analyses as described below. This study did not assess sex differences.

### Feeding trial design

Zebrafish from 7 to 21 dpf were fed three times a day alternating commercial dry food (Zebrafish Management Ltd) and brine shrimp (Zebrafish Management Ltd). Fish were then fed for another week three times a day with the dry regular phosphorus (RP) diet until 28 dpf. The nutrition trial started at 28 dpf: WT and transgenic fish were randomly divided in three groups (*n* = 12 per group), grown in identical tanks with a density of 10 fish/L, and fed three times a day with a low-P (LP) diet, an RP diet, and a high-P (HP) diet, respectively, for two months, as previously described.^24^

In the second nutritional trial, starting from 28 dpf, WT fish (*n* = 36) were fed a LP diet for two months as described above, then were randomly divided in three groups (*n* = 12 per group) and fed either LP (LP–LP group), RP (LP–RP group), or HP (LP–HP group) diet for 6 weeks. A control group (*n* = 12) was fed RP throughout.

### Diet composition

Custom made zebrafish were formulated by SimplyFish AS (Norway) to have a total P content of 0.5%, 1.0%, and 1.5%, termed LP diet, RP, and HP diet, respectively. Monoammonium phosphate (NH₄H₂PO₄, MAP) was used as dietary inorganic P supplement. Details of diet composition are described in detail in Cotti et al.^24^

### Quantitative real-time PCR

Following dissection of LP, RP, and HP zebrafish (*n* = 4 per group), the vertebral column excluding the caudal complex was cleaned from the muscles, the neural tube, and the blood vessels and stored in 1 mL TRIzol reagent (Invitrogen) at −80°C. The dissected vertebral column was homogenized through high-speed shaking with 3 mm Tungsten Carbide beads (Qiagen) using a TissueLyser II (Qiagen). Total RNA was isolated following manufacturer’s instructions and quantified by Nanodrop ND-1000 spectrophotometer (Thermo Fisher). About 1 μg of total RNA was treated with genomic DNase and retro-transcribed into cDNA using PrimeScript RT Reagent Kit (Takara Bio Inc.) following manufacturer’s instructions. Quantitative real-time PCR (qPCR) was performed in a reaction volume of 10 μL containing PowerUp SYBR Green Master Mix (Applied Biosystems), validated qPCR primers (BLAST, Table S1) and cDNA using the QuantStudio3 Real-Time PCR System (Thermo Fisher). An initial 2 min step at 50°C was followed by denaturation of 2 min at 95°C and amplification (10 s at 95°C, 30 s at 58°C, 40 cycles). The annealing temperature was 58°C for all the primers used. qPCR efficiency and specificity were determined per primer set by melting curve analysis. Samples were run in triplicate, *ef1a* was employed as housekeeping gene, given that it is one of the most stable genes with a low degree of variability.^28^ The differential expression was calculated according to the 2^−ΔΔCt^ method.^29^

### Histology

**Figure 1:**
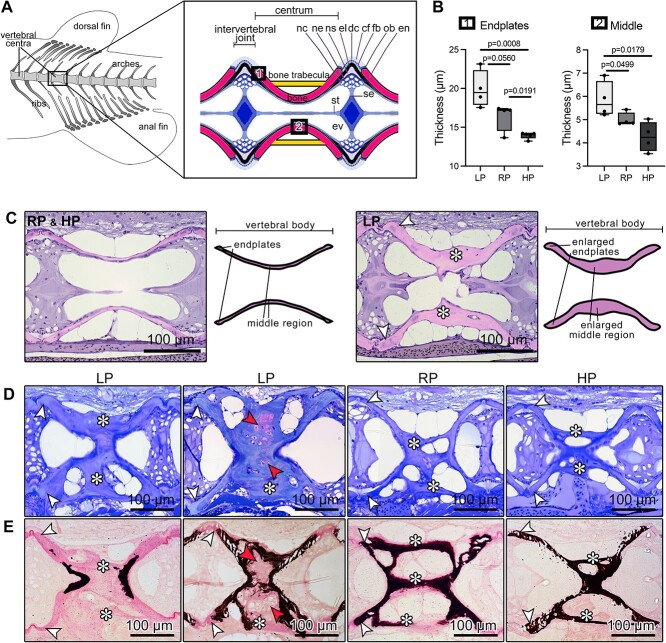
Bone formation increases and bone mineralization is arrested following LP diet. (A) Scheme of the vertebral column of an adult zebrafish indicating the location of the vertebral centra used for analysis. The box shows a schematic of the midline sagittal plane of a vertebral body centrum and two intervertebral joints. Osteoblasts (ob) deposit new bone matrix at the vertebral body endplates (en), i.e. the bone growth zones, which are connected by intervertebral ligaments. From inside to outside, the ligaments consist of the notochord sheath (ns, a collagen type II layer secreted by the cells of the notochord epithelium, ne), the outer elastin layer (el), and dense collagen type I fiber bundles (dc) produced by fibroblasts (fb). The collagen type I fiber bundles (cf) continue in the bone of the vertebral body endplates (en) as Sharpey fibers. The notochord is composed of vacuolated notochord cells (nc) and extracellular vacuoles (ev). Condensed notochord cells constitute the notochord septum (se) and the notochord strand (st). Boxes show locations where the bone thickness was measured, i.e. endplates (1) and middle region of the autocentrum (2). (B) Bone thickness measurements at vertebral endplates and middle region of the autocentrum from LP, RP, and HP animals aftertwo months of dietary treatment. LP zebrafish have thicker structures compared to RP and HP animals (see also Table S2). Values are reported with Box and Whisker plots: the midline in each box is indicative of the median, whereas min and max values are shown with whiskers. Individual data points and *P* values are shown. (C) Sagittal demineralized histological sections stained with elastin staining and schematics showing vertebral bodies from RP (representative also of HP) and LP animals. LP zebrafish have enlarged vertebral body endplates (white arrowheads) and middle region (asterisks) compared to RP and HP. (D) Toluidine blue stained sagittal sections of vertebral bodies from LP, RP, and HP show that vertebral endplates (white arrowheads) and trabecular bone (asterisks) are considerably thicker in LP animals in comparison with controls and HP fish. Red arrowheads show chondroid bone. (E) Von Kossa/Van Gieson stained non-demineralized sections show a similar sectional plane as in D. The additional bone formed under the LP diet conditions is mostly non-mineralized at vertebral endplates (white arrowheads) and trabecular bone (asterisks) compared to RP and HP. Non-mineralized bone: pink; mineralized bone: black. Some vertebral bodies from LP individuals present enlarged (asterisks) bone trabeculae characterized by the presence of chondroid bone (red arrowheads), which is largely non-mineralized as demonstrated by Von Kossa/Van Gieson staining.

Specimens were fixed for 24 h in 2.5% PFA, 1.5% glutaraldehyde, 0.1 M sodium cacodylate buffer (pH 7.4), and 0.001% CaCl_2_ at 4°C, decalcified in 0.1 M EDTA for 14 d at 4°C and embedded in glycol methacrylate.^24^ Sagittal or transverse 2 μm sections were cut on a Microm HM360 (Marshall Scientific) automated microtome and were stained with toluidine blue (0.5% toluidine blue, 1% Na_2_B_4_O_7_ in demineralized H_2_O (dH_2_O), pH 9 for 15 s) or with elastin staining,^30^ rinsed in dH_2_O, and mounted with DPX. Images were acquired using an Axio Imager-Z1 microscope (Carl Zeiss) equipped with an Axiocam 503 color camera (Carl Zeiss) using the ZEN software (Carl Zeiss). Bone structure histomorphometry was analyzed on images of toluidine blue stained sagittal sections of the middle plane of the vertebral column according to an established protocol.^25^ Thickness of bone structures in all the four endplates of the centrum on the section (anterior and posterior, dorsal and ventral, respectively) and in the middle region (dorsal and ventral) of 5 to 10 vertebral centra per specimen (LP *n* = 4, RP *n* = 4, HP *n* = 5) was measured using Fiji (NIH) (see Figure 1A for location). The mean values per location per specimen were considered for analysis.

For mineral detection on histological sections, specimens were fixed as described above and embedded in glycol methacrylate without decalcification. Sagittal or transverse sections of 2 μm were stained according to the Von Kossa/Van Gieson protocol.^30^ Images were acquired using an Axio Imager-Z1 microscope (Carl Zeiss) equipped with a 5MP CCD camera using the ToupView software (ToupTek Photonics). Sagittal sections of the middle plane of the vertebral column were used for quantification analysis of 7 to 10 vertebral endplate growth zones per specimen (RP–RP control *n* = 3, LP–LP *n* = 4, LP–RP *n* = 4, LP–HP *n* = 4). For each specimen, the region of interest corresponded to a rectangle with a defined length (one-third of the total vertebral length) and height (one-quarter of the total vertebral height), centered in the middle of the intervertebral ligament. Within the region of interest, the total bone area and the mineralized bone area were measured using Fiji (NIH) and used to calculate the following parameters: (1) percentage of mineralized bone, calculated as the ratio between the mineralized bone area and the total bone area, (2) the total bone area normalized to the vertebral length. The mean values per specimen were considered for analysis.

**Figure 2:**
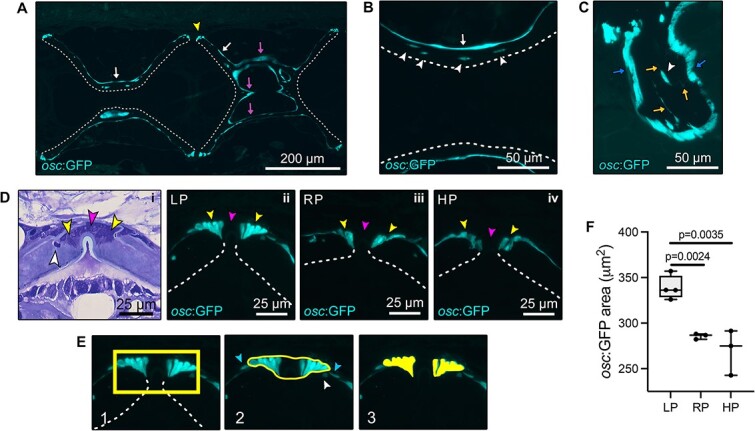
Expression of osteocalcin in the vertebral column assessed in transgenic *osc:*GFP zebrafish line fed LP, RP, HP for two months. (A–C) Independent from dietary P intake, osteocalcin is expressed by osteoblasts at vertebral body endplates (yellow arrowhead, A) and by bone lining cells at the bone surface (white arrows, A,B), the trabecular bone (magenta arrows, A) and arches (blue arrows, C). Dashed lines indicate the inner bone surface. Osteocytes (white arrowheads in B and C) are *osc:*GFP positive. The osteocytic canalicular network is also labeled (yellow arrows, C). (D) Toluidine blue stained zebrafish vertebral endplates (i) showing osteoblasts (yellow arrowheads), fibroblasts (magenta arrowheads), and osteocyte (white arrowhead). Osteoblasts (yellow arrowheads) from LP (ii), RP (iii), and HP (iv) zebrafish are *osc:*GFP positive, but fibroblasts (magenta arrowheads) are *osc:*GFP negative. (E) Selection of the area used for quantification of the *osc:*GFP-positive area occupied by osteoblasts at vertebral body endplates, considering only osteoblasts and excluding osteocytes (white arrowhead) and bone lining cells (light blue arrowheads). (F) LP zebrafish have a significantly increased *osc:*GFP signal area, i.e. the area occupied by GFP-positive osteoblasts, at vertebral endplates compared to RP and HP. Values are reported with Box and Whisker plots: the midline in each box is indicative of the median, whereas min and max values are shown with whiskers. Individual data points and *P* values are shown.

After two months of dietary treatment, *osc*:GFP zebrafish were euthanized by tricaine overdose and kept in the dark during the following steps. To preserve and visualize GFP on sections, we followed an established protocol.^31^*osc*:GFP zebrafish were then embedded in glycol methacrylate.^24^ Sagittal sections of 3 μm were cut on a Microm HM 360 (Marshall Scientific) automated microtome. Images were acquired using an Axio Imager-Z1 microscope (Carl Zeiss) using the ZEN software (Carl Zeiss). The sagittal section at the exact middle plane of the vertebral column of each specimen was used for quantification of the fluorescence-positive area occupied by osteoblasts in the vertebral endplate growth zones of the caudal region (a total of 20 growth zones were analyzed per fish, LP *n* = 4, RP *n* = 3, HP *n* = 3). Osteocytes (embedded in the bone matrix) and bone lining cells (flat, elongated cells on the outer surface of bone not located in the vertebral body endplates region) were not considered in the analysis (Figure 2E). The ZFBONE ImageJ toolset^32^ for Fiji (NIH) was used for quantification. The mean values per specimen were considered for analysis.

### Enzyme histochemistry

For demonstration of tartrate-resistant acid phosphatase (TRAP), samples (RP–RP control, LP–LP, LP–RP, LP–HP, *n* = 1 per group) were fixed in 4% PFA in 1× PBS, pH 7.4, for 1 h at RT and decalcified in 4% PFA, 10% EDTA, pH 7.4 for 14 d at 4°C, then embedded in glycol methacrylate.^24^ Transverse 5 μm sections were cut on a Microm HM360 (Marshall Scientific) automated microtome and demonstration of TRAP was performed as described in Cotti et al.^25^

### Alizarin red S

Zebrafish (LP–LP, LP–RP, LP–HP, RP–RP, *n* = 3 per group) were euthanized by tricaine overdose, fixed for 24 h in 4% paraformaldehyde (PFA) in 1× PBS at 4°C, and were stained as described in Cotti et al.^25^ The vertebral column was then dissected and imaged using an Axio Imager-Z1 microscope with DIC illumination (Carl Zeiss).

### Micro-computed tomography

For micro-CT, LP–LP, LP–RP, LP–HP, and RP–RP controls (*n* = 3 per group) were fixed in 4% PFA in 1× PBS, pH 7.4, for 24 h at 4°C. After rising in 1× PBS, the samples were moistened in PBS, wrapped in parafilm, and placed in a cylindrical sample holder suitable for high resolution scanning with a SkyScan1276 Microtomograph (Bruker). Scans of the caudal region of the vertebral column were acquired at a 3.38 μm voxel size, with a 41 kV voltage and 72 μA current. A 0.25 mm aluminum filter was used. Calibration rods of known mineral density (0.25 and 0.75 g/cm^3^ hydroxyapatite, 2 mm diameter) were scanned under the same experimental conditions applied to the zebrafish samples for quantification of the density of zebrafish vertebral bodies. NRecon software (Bruker) was used for scan reconstructions, for which Gaussian smoothing, defect pixel masking, beam hardening correction, and attenuation range were kept constant for all the samples and phantoms. Post-alignment fine tuning and ring artifact reduction were adapted to each scan. The volume of interest, including the first three caudal vertebral bodies, was first selected using DataViewer software (Bruker) and the analysis was performed using CTAn software (Bruker). The ROIs, i.e. the vertebral centra of the first three caudal vertebral bodies, were selected semiautomatically, applying initially a manual ROI enclosing each central vertebra and subsequently an in-house developed task list based on multiple steps of global thresholding, to segment only the mineralized bone tissue and exclude soft tissues and non-mineralized bone as well. The selected ROI were used to calculate BMD (defined as g/cm^3^ of calcium hydroxyapatite, calibrated against the density scales of the attenuation coefficient) and the bone volume (mm^3^). Data obtained refer only to the mineralized parts of the ROI, as non-mineralized bone was not segmented.

For 3D visualization of the vertebral body analyzed, the volume of interest was reconstructed using Amira 3.1.1 software (Thermo Fisher) by segmentation applying a constant greyscale-threshold to all samples, to visualize the mineralized parts of the vertebral bodies and arches, as previously described.^24^

### Statistical analysis

Quantitative variables are expressed as mean ± standard deviation. Statistical analysis was performed using Past 4.04 software.^33^ Multiple groups’ differences were based on one-way ANOVA followed by pairwise comparisons based on Student’s *t*-test. Differences in gene expression were evaluated by means of Student’s *t*-test followed by Bonferroni correction. A *P* value less than 0.05 was considered significant.

## Results

### Low dietary P intake increases bone matrix formation and arrests mineralization

Feeding zebrafish a LP diet for two months increases bone matrix formation and reduces mineralization, strengthening our previous findings (Figure 1).^24^ In view of a translational approach, a quantitative assessment of the effects of the P diets on bone tissue and cells is needed. Thus, we further analyze here the vertebral column of zebrafish that received LP, RP (control), or HP diet. The regions of interest correspond to the vertebral body endplates and the middle region of the vertebral bodies analyzed on sagittal histological sections of the vertebral column (Figure 1A). In zebrafish, and teleosts in general, vertebral bodies consist of the mineralized notochord sheath (chordacentrum) and bone that forms around the notochord sheath. Perichordal bone formation creates an hourglass-shaped double cone (autocentrum). The margins of opposing cones are also designated as vertebral body endplates and in zebrafish are connected with thin, plate-like bone trabeculae^25^ (Figure 1A). Histomorphometry was used to quantify the bone thickness at the vertebral body endplates and the middle region of the autocentrum (Figure 1B). In LP animals, bone thickness is significantly increased in both regions, with a considerable increase in the middle part of the vertebral bodies compared to RP and HP animals (Figure 1B and C**,**Table S2). Conversely, HP zebrafish have thinner bone structures compared to LP and RP animals (Figure 1B and C**,**Table S2). Mineralization arrests upon LP diet administration, as demonstrated by Von Kossa/Van Gieson staining. Some LP animals with enlarged bone trabeculae developed chondroid bone, i.e. chondrocyte-like cells embedded in bone-like matrix^34^^,^^35^ (Figure 1D and E), a common character of fast-growing bone tissue.^36^ In RP animals, instead, vertebral body endplates and bone trabeculae are fully mineralized, and bone is covered by a typical, thin, non-mineralized osteoid layer. In HP animals, the osteoid layer is not detectable (Figure 1E).

Together, these quantitative data based on histomorphometry strengthen the previous qualitative observations and clearly demonstrate that LP conditions significantly increase bone thickness and reduce mineralization at the level of the vertebral column, compared to RP and HP diet.

### Osteocalcin is upregulated under LP conditions

Expression of osteocalcin, the most abundant non-collagenous protein produced by osteoblasts, was evaluated using *osc*:GFP transgenic zebrafish line fed the three different P diets. In animals from all three dietary groups, osteocalcin is expressed in osteoblasts, bone lining cells, osteocytes, and in the osteocyte canalicular network (Figure 2A–C). In the growth zone of the vertebral body endplates, osteoblasts strongly express osteocalcin, as do bone lining cells, osteoblasts, and osteocytes located in the central region of the autocentrum (Figure 2B), the arches (Figure 2C), and the bone trabeculae that connect the vertebral body endplates (Figure2A). The osteocyte canalicular network is also clearly *osc*:GFP positive and both osteocyte-osteocyte and osteoblast-osteocyte connections are visible (Figure 2C). In contrast, fibroblasts in the intervertebral ligament are negative for osteocalcin in all dietary groups (Figure 2D). Quantification of the area occupied by GPF-positive osteoblasts (*osc*:GFP-positive area), at the vertebral body endplates (Figure 2D and E), yielded a significantly increased value in LP zebrafish compared to RP and to HP zebrafish (Figure 2F).

### Upregulation of bone matrix formation and mineralization-related genes in LP animals

**Figure 3:**
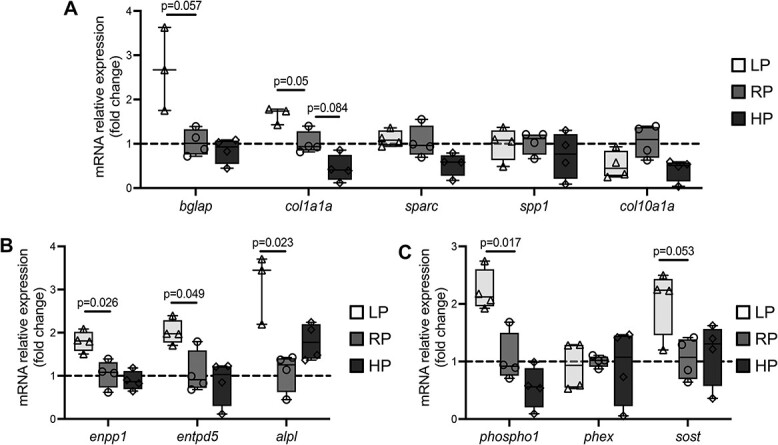
qPCR results. (A) Expression of the osteoblast-related markers osteocalcin (*bglap*) and collagen type I (*col1a1a*) is upregulated in LP animals compared to controls. Other osteoblast markers, i.e. osteonectin (*sparc*), osteopontin (*spp1*), and collagen type X (*col10a1a*), do not show differential expression between the three dietary groups. (B) Expression of the mineralization-related markers ectonucleotide pyrophosphatase phosphodiesterase 1 (*enpp1*), ectonucleoside triphosphate/diphospho-hydrolase 5 (*entpd5*), and tissue non-specific alkaline phosphatase (*alpl*) is significantly upregulated in LP zebrafish compared to controls. (C) Expression of the P-associated markers phosphatase orphan 1 (*phospho1*) and sclerostin (*sost*) is upregulated in LP zebrafish compared to controls. Phosphate regulating gene with homologies to endopeptidases on the X chromosome (*phex*) is not differentially expressed. In all panels, values are reported with Box and Whisker plots: the midline in each box is indicative of the median, whereas min and max values are shown with whiskers. Individual data points and *P* values are shown.

The upregulation of GFP osteocalcin signal detected in the transgenic zebrafish line fed LP was confirmed by qPCR analysis of *bglap*. The expression of *bglap*, marker for late osteoblasts differentiation, is significantly increased by almost a 3-fold in LP zebrafish compared to RP and HP (Figure 3A).

The expression of other late osteoblast markers associated with bone matrix formation, i.e. collagen type I (*col1a1a*), osteonectin (*sparc*), osteopontin (*spp1*), and the early osteoblast marker collagen type X (*col10a1a*) was also evaluated by qPCR (Figure 3A). *col1a1a* is significantly upregulated in LP animals compared to RP, and *col1a1a* shows a trend toward downregulation in HP compared to RP. This matches the histomorphometry data about increased bone matrix apposition in LP and thinner bone structures in HP. All the other markers analyzed do not show differential expression between the three dietary groups (Figure 3A).

The expression of mineralization-related genes was also evaluated by qPCR, namely the gene encoding the ectonucleotide pyrophosphatase phosphodiesterase 1 (*enpp1*) that catalyzes the production of the mineralization inhibitor pyrophosphate (PPi), and the genes encoding two enzymes that hydrolyze extracellular PPi: ectonucleoside triphosphate/diphospho-hydrolase 5 (*entpd5*) and tissue non-specific alkaline phosphatase (*alpl*). Under LP conditions, *enpp1*, *entpd5*, and *alpl* show significantly increased expression compared to RP. No differential expression is detected between RP and HP (Figure 3B). Low dietary P intake also upregulates the expression of phosphatase orphan 1 (*phospho1*), an intracellular phosphatase that releases P from phosphorylated substrates, and sclerostin (*sost*) that regulates not only osteoblastic bone formation, but also calcium and P homeostasis.^37^ No differences are found between RP and HP (Figure 3C). No differential expression among the three dietary groups is detected for *phex*, the phosphate regulating gene with homologies to endopeptidases on the X chromosome (Figure 3C).

To assess whether the increased expression of *alpl* in LP animals at the transcription level is also accompanied by an increased activity of alkaline phosphatase (ALP), an enzyme histochemistry assay was performed. In all dietary groups, ALP activity is detected at the vertebral body endplates in osteoblasts, fibroblasts, and cells of the notochord epithelium (Figure S1A). Only in LP zebrafish, ALP activity is also found in bone lining cells of bone trabeculae (Figure S1B) and the vertebral centrum (Figure S1C). ALP activity is also detected in cartilage-like cells of the chondroid bone found in some LP vertebral bodies (Figure S1D–E).

Together, these data demonstrate upregulation of key genes of bone matrix formation and mineralization under LP dietary conditions.

### Bone matrix deposited during low dietary P intake retains the ability to mineralize

**Figure 4:**
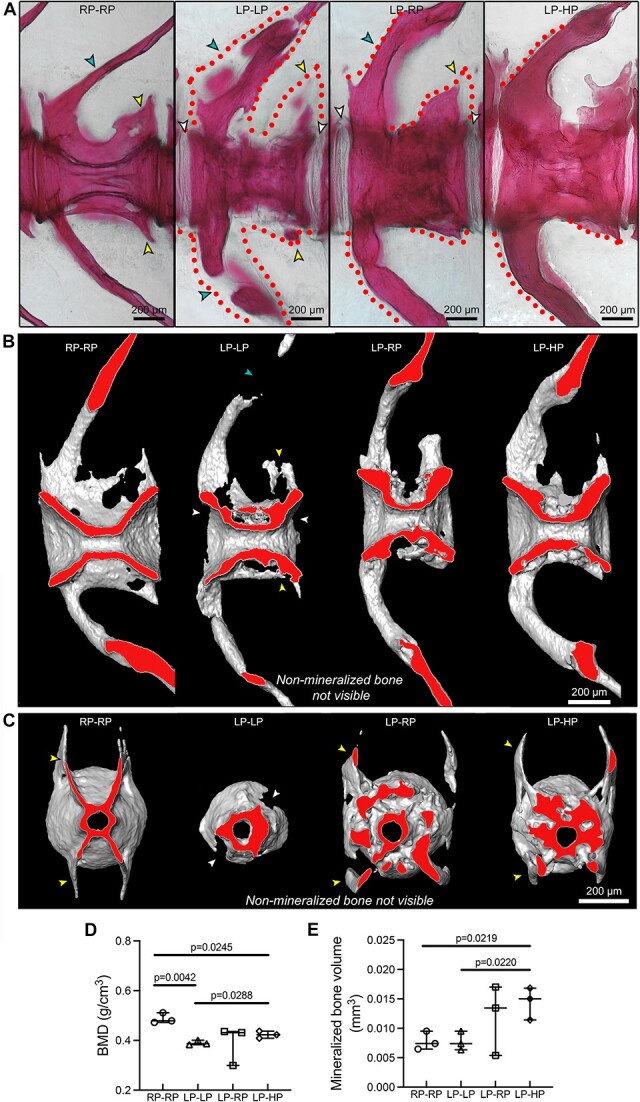
3D assessment of the LP-driven increase in bone matrix volume that subsequently mineralizes with sufficient-P intake. (A) Whole-mount Alizarin red S staining shows that LP–LP vertebrae present several non-mineralized (dotted lines, negative for the staining) bone structures compared to controls (RP–RP), i.e. the vertebral body endplates (white arrowheads), neural and haemal arches (turquoise arrowheads), and zygapophysis (yellow arrowheads). These structures are almost fully mineralized when sufficient dietary P is provided, as shown in LP–RP and LP–HP zebrafish. Virtual sagittal (B) and cross (C) sections from micro-CT-based reconstructions of vertebral bodies show the mineralization portion of bone only. The virtual sectional plane labeled in red shows the increased amount of bone around the vertebral centrum in LP–LP, LP–RP, and LP–HP zebrafish compared to controls. Gaps in bone structures are non-mineralized bone structures that are not visible with micro-CT. Arrowheads as in A. (D and E) Micro-CT analysis of the mineralized parts of bone. (D) BMD is reduced in LP–LP and LP–HP zebrafish compared to controls. (E) A dietary P-dependent increase in the bone volume (mineralized bone only) is demonstrated in LP–HP compared to control and LP–LP zebrafish. Values are reported with Box and Whisker plots: the midline in each box is indicative of the median, whereas min and max values are shown with whiskers. Individual data points and *P* values are shown.

After two months of LP diet, LP zebrafish were divided into three groups and fed LP (LP–LP), RP (LP–RP), or HP (LP–HP) diets for 6 weeks up to the age of 4.5 months. A control group was fed RP diet throughout (RP–RP). Whole mount staining with Alizarin red S shows increased bone, both mineralized (red) and non-mineralized (translucent), deposited around the vertebral bodies as well as at neural and haemal arches in all animals with LP dietary history compared to RP–RP controls (Figure 4A). Bone in LP–LP animals shows a patchy mineralization. However, sites of active bone apposition remain non-mineralized, including the vertebral body endplates, the neural and haemal arches, and growing bone trabeculae. In LP–RP the rim of bone at growth sites is non-mineralized, whereas in LP–HP animals these bone elements are almost fully mineralized (Figure 4A).

**Figure 5:**
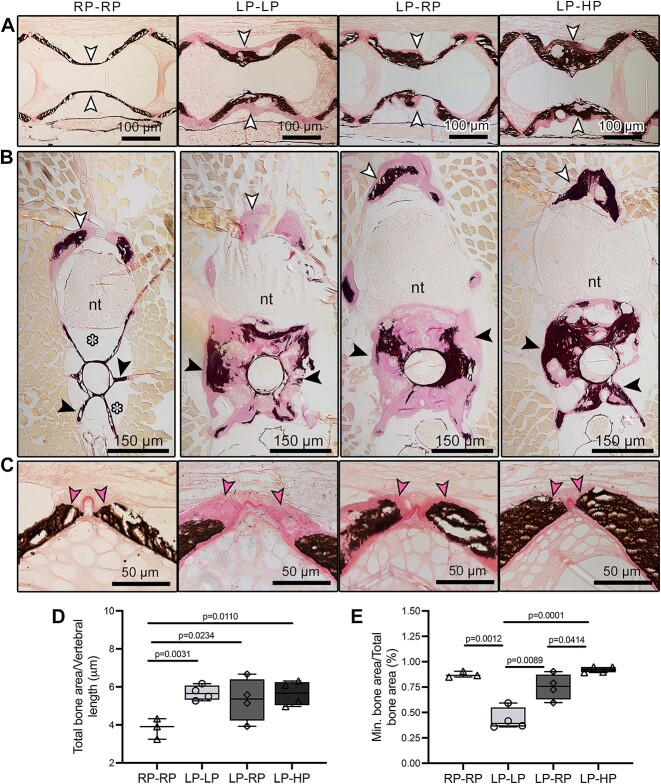
2D assessment of the LP-driven increase in bone matrix volume that subsequently mineralizes with sufficient-P intake. Sagittal (A and C) and cross (B) non-demineralized histological sections of the vertebral column stained with Von Kossa/Van Gieson. Compared to controls, LP–LP, LP–RP, and LP–HP animals show an increase in bone volume in the middle region of the vertebral centrum (white arrowheads in A, black arrowheads in B), at the arches (white arrowheads in B), and at the vertebral body endplates (pink arrowheads, C). In LP–LP animals, the amount of non-mineralized bone matrix (pink staining) is larger compared to that in LP–RP and LP–HP animals, where the bone structures are mineralized (black staining). In all zebrafish with LP dietary history, the enlarged bone fills the space occupied by fat in RP–RP (white asterisks). Nt: neural tube. (D and E) Histology-based measurements. (D) The total bone area (mineralized and non-mineralized) at the vertebral body endplates, normalized to vertebral body length, is significantly increased by 1.5-fold in LP–LP, LP–RP, and LP–HP zebrafish compared to controls (RP–RP). (E) At the vertebral body endplates, the mineralized bone area divided by the total bone area, used as indicator of mineralization, shows that bone mineralization depends on dietary P, and non-mineralized bone generated during the LP period can resume mineralization to an extent similar to controls when sufficient-P is provided with the diet. Values are reported with Box and Whisker plots: the midline in each box is indicative of the median, whereas min and max values are shown with whiskers. Individual data points and *P* values are shown.

Vertebral columns from all dietary groups were also analyzed by micro-CT, which allows to visualize only the mineralized parts of bone. Non-mineralized bone structures, especially in LP–LP zebrafish, are therefore not visualized with micro-CT, whereas they are visible on histological sections (Figure 5). This helps in appreciating the striking increase of mineralized bone volume in LP–RP and LP–HP animals compared to RP–RP controls. Both on sagittal and cross virtual sections obtained from micro-CT-based 3D reconstructions, the vertebral body endplates, the neural and haemal arches (Figure 4B), and the trabecular bone (Figure 4C) appear strongly enlarged compared to RP–RP controls. On the contrary, bone elements of LP–LP vertebral bodies appear poorly structured on micro-CT reconstruction, but this is not surprising given the low level of mineralization. Bone matrix is present, simply it does not mineralize (yet), as shown in stained whole mounts (Figure 4A).

Micro-CT scans of the first three caudal vertebral bodies in each dietary group were then analyzed to quantify the BMD and the bone volume. Data refer to the mineralized parts of the vertebral centrum only. Arches and zygapophysis were excluded from the analysis. BMD is significantly reduced in LP–LP and LP–HP animals compared to RP–RP controls, as expected given the LP dietary history (statistical significance is not reached in LP–RP, likely due to the high standard deviation). Interestingly, BMD values show a tendency toward increased values in a dietary-P-dependent manner (Figure 4D). This suggests that BMD values can resume control values when sufficient dietary P is provided. Still, interestingly LP–HP have a statistically lower BMD compared to RP–RP controls.

Micro-CT analysis demonstrates increased bone volume, that is, mineralized bone volume, in LP–HP vertebral bodies compared to both RP–RP and LP–LP in a dietary P-dependent manner. Bone volume displays no differences between RP–RP and LP–LP (Figure 4E). This is not surprising, considering the enlarged bone trabeculae deposited around the centrum in LP–LP vertebrae, which are also partially mineralized (Figures 4 and 5).

To further demonstrate that LP conditions increase mineralized and non-mineralized bone volume in zebrafish regardless of the diet received after the LP period, sagittal and cross non-demineralized sections stained with Von Kossa/Van Gieson from controls (RP–RP), LP–LP, LP–RP, and LP–HP were used (Figure 5). The increase in the amount of bone is striking in LP–LP, LP–RP, and LP–HP compared to controls, both at the vertebral body endplates and central region of the vertebral centrum (Figure 5A and B). In LP–LP animals, the amount of non-mineralized bone matrix at both areas is larger compared to LP–RP and LP–HP animals (Figure 5A and B), as also shown at higher magnification in the vertebral endplates (Figure 5C). At vertebral body endplates locations, midline sagittal sections of the vertebral column were used to quantify the total bone area normalized to vertebral length (Figure 5D). The total normalized bone area is significantly increased by 1.5-fold in LP–LP, LP–RP, and LP–HP compared to controls. Interestingly, there is no difference in the total (sum of mineralized and non-mineralized) normalized bone area among LP–LP, LP–RP, and LP–HP, confirming that the LP period common to the three groups caused the increase in bone volume. At the same vertebral endplates, the mineralization extent in LP–LP, LP–RP, and LP–HP compared to controls, was quantified as the mineralized bone area over the total bone area (Figure 5E). LP–LP animals have drastically reduced mineralization (less than 50%) compared to controls. LP–RP vertebrae show an increase in the mineralization extent compared to the control values, whereas the LP–HP group has a higher mineralization extent with respect to the controls. All vertebral bodies in all examined fish from each dietary group show the phenotype in a consistent manner throughout the vertebral column (Figure S2). The substantial amount of new bone that is generated does not extend into the intervertebral joints. Enlarged bone trabeculae are not subjected to increased bone resorption (Figure S3).

These data further confirm that non-mineralized bone produced under LP conditions retains the ability to mineralize when adequate dietary P is provided. The result is a substantial increase of the mineralized vertebral body bone volume compared to control animals.

## Discussion

The present study demonstrates a strategy to significantly increase bone volume in zebrafish by alternating levels of dietary P intake. We deepen the current knowledge on the effects of an LP diet on bone tissue and cells^22^^,^^24^ with quantitative histomorphometry data and molecular analyses. In addition, we present a means to turn the increased non-mineralized matrix into mineralized bone in zebrafish. Thus, in this study, a LP dietary intake triggers increased non-mineralized bone matrix formation, which is capable of mineralization upon sufficient dietary P intake, hence resulting in an enormous increase of the mineralized bone at the end of the experiment.

### Increased bone matrix formation is accompanied by the upregulation of key osteoblast markers under LP conditions

The increased formation of non-mineralized bone matrix in response to a limited period of low dietary P intake confirms earlier studies on teleosts and mammals.^22^^,^^24^^,^^38^ The LP zebrafish bone has characters of hypophosphatemia and osteomalacia, given that bone from hypophosphatemic human patients and mice shows greatly increased osteoid surfaces, volume, and thickness.^37^^,^^39^^,^^40^ However, the LP diet stimulates the accumulation of non-mineralized bone matrix without causing severe skeletal malformations and the adverse symptoms commonly associated to hypophosphatemia and osteomalacia.^41^ Moreover, it is interesting to note that a phenotype with increased amounts of non-mineralized bone matrix commonly occurs in human teenagers. In this period, fast-growing bone can be relatively under-mineralized, yet without malformations.^42^^,^^43^

The LP zebrafish, human adolescents, and osteomalacia patients (considering only the common aspect of increased bone matrix formation) present similar expression of biochemical markers as indicators for bone matrix formation.^42^^,^^44^ In adolescents and patients with osteomalacia, and mouse models, the excess production of bone matrix is accompanied by elevated levels of the circulating bone formation marker P1NP, the N-terminal propeptide of collagen type I.^37^^,^^45^^,^^46^ P1NP is removed by specific enzymes before the collagen molecule forms, thus it reflects the osteoid formation by osteoblasts prior to mineralization and it is directly related to the amount of newly formed collagen matrix in bone.^37^^,^^47^ Although analogous blood analysis in zebrafish is not possible, histology measurements and gene expression data from LP zebrafish clearly demonstrate upregulation of collagen type I (*col1a1a*).

Likewise, serum osteocalcin is another commonly used marker for bone formation^48^ and can precisely reflect the status of bone formation, including the activity of osteoblasts and the rate of bone mineralization.^47^^,^^49^ Osteocalcin was found elevated in blood from human adolescents, osteomalacia patients, and mice, in wild-type mice fed a P-deficient diet,^37^^,^^40^^,^^46^^,^^47^^,^^50^ all conditions for which increased bone formation is a common character. Similarly, an osteocalcin transgenic zebrafish line and qPCR data demonstrate upregulation of *bglap* in zebrafish fed LP diet. These observations support the similarity between the LP zebrafish model and human conditions of increased bone matrix production, both in health and disease.

### The upregulation of mineralization-related genes ensures the mineralization capability of the bone matrix

Reduced mineralization levels characterize the LP zebrafish bone, as well as bone from patients and mouse models that suffer from osteomalacia.^37^^,^^40^^,^^50^^,^^51^ Patients and mice have low plasma P concentrations.^37^^,^^50^^,^^51^ Although we do not have plasma P levels from zebrafish, based on LP plasma levels found in Atlantic salmon^26^ and mice^50^ fed a LP diet, it is reasonable to assume that LP diet reduces P concentrations also in zebrafish blood. Low plasma P levels trigger a response committed to mitigate the deficiency of the substrate (P) required for bone mineralization. Human adolescents as well as patients and mouse models of hypophosphatemia show elevated concentrations of blood ALP.^37^^,^^40^^,^^45–47^ This enzyme breaks down the mineralization inhibitor pyrophosphate (PPi) and liberates P. Similarly, LP conditions in zebrafish increase *alpl* mRNA levels, encoding for the tissue non-specific Alp. Other genes encoding for enzymes relevant for mineralization and expressed by osteoblasts are upregulated in LP zebrafish, that is, *enpp1*, *entpd5*, and *phospho1.* The upregulation of genes important for mineralization in the LP zebrafish suggest an attempt to rescue the lack of mineralization. On the one hand, the upregulation of *entpd5* and *phospho1* could directly liberate P from their substrates. The upregulation of *enpp1*, that provides the substrate (PPi), and *alpl*, that hydrolyzes the mineralization inhibitor PPi, could offer additional P for mineralization. In our study, LP zebrafish show also increased expression of *sost*, encoding for sclerostin. Likewise, circulating sclerostin levels are elevated in patients and mice affected by hypophosphatemia,^37^^,^^52^^,^^53^ and sclerostin mRNA was upregulated in the femur of the *HYP* mouse.^54^ Different from mammals, initial studies showed that in zebrafish, *sost* is primarily expressed by osteoblasts rather than osteocytes.^55^ Thus, apart from regulating osteoblastic bone formation, sclerostin may regulate calcium and P homeostasis.^37^^,^^54^

**Figure 6:**
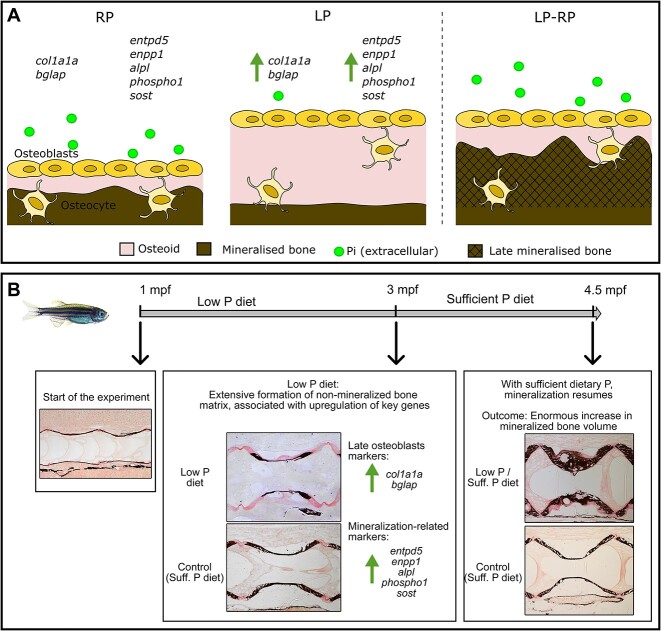
Schematic view of the effects of dietary P intake on bone formation and mineralization. (A) In presence of regular dietary P (RP), the non-mineralized bone matrix secreted by osteoblasts, named osteoid, mineralizes after deposition. When low dietary P (LP) is provided, bone matrix production is stimulated, accompanied by the increased expression of the osteoblast specific markers collagen type I (*col1a1a*) and osteocalcin (*bglap*). Under LP conditions, genes important for bone mineralization are upregulated, such as the ectonucleotide pyrophosphatase phosphodiesterase 1 (*enpp1*), the ectonucleoside triphosphate/diphospho-hydrolase 5 (*entpd5*), the tissue non-specific alkaline phosphatase (*alpl*), the phosphatase orphan 1 (*phospho1*), and sclerostin (*sost*). Thus, under LP conditions, osteoblasts increase the production of non-mineralized bone matrix and the expression of genes required for mineralization. When sufficient dietary P is provided (LP–RP), the mineralization of the non-mineralized matrix resumes, resulting in increased mineralized bone volume. (B) Summary of the main results of the zebrafish dietary P experiment. The LP diet stimulates bone matrix formation first associated to upregulation of key genes. Upon sufficient dietary P administration, newly formed bone matrix mineralizes, resulting in enormous increase in mineralized bone volume.

Based on the upregulation of genes important for bone mineralization and mineral homeostasis, it is reasonable to assume that the organic matrix produced under LP conditions is ready to mineralize. Still, mineralization does not occur until adequate P levels are provided (Figure 6A).

### The alternation of LP and sufficient-P generates a lasting increased bone volume

The low dietary P condition leads to a substantial increase in the formation of non-mineralized bone. The increase in bone matrix production is consistent throughout the vertebral column and is not only confined to locations of bone formation related to length increase, i.e. the vertebral body endplates, or to locations of increased mechanical loading such as the caudal part of the vertebral column. It also occurs at the periosteal surfaces of the neural and haemal arches, in the trabeculae, and in the central region of the autocentra. Prolonged LP conditions generate a thick bone matrix that is largely non-mineralized, as expected. Consistently, micro-CT analysis shows a significant reduction in BMD in the LP–LP animals compared to controls and the LP–HP dietary group. This indicates that, on the one hand, the large non-mineralized bone derived from the former LP period resumed mineralization. On the other hand, it is likely that the recently mineralized bone has slightly improved mechanical properties, given that mineralization is resumed in LP–HP but with a reduced amount of minerals (lower BMD). BMD is a measure of the bone mineral content (of the mineralized parts of bone) and was, in this study, performed only on the mineralized parts of the vertebral bodies. Interestingly, LP–LP animals have reduced BMD values in the mineralized regions of the autocentra, suggesting reduced mineral content in those mineralized areas, consistent with the reduced elastic modulus and hardness demonstrated by nanoindentation assessment of LP bone mechanical properties.^24^ Low BMD also typifies under-mineralized bones (osteomalacia) in humans, where it reflects the presence of large amounts of non-mineralized osteoid rather than the reduction of bone matrix volume.^51^ Irrespective of whether the disease is due to nutritional deficiencies or to genetic mutations,^56^ osteomalacia is generally accompanied by decreased P in the serum of patients.^51^ Treatment of osteomalacia in humans consists of adequate calcium intake and vitamin D and phosphate oral supplementations, which promote the non-mineralized matrix to become mineralized and favor the increase of BMD values.^51^^,^^57^ Likewise, increasing the P plasma levels by dietary means in teleosts,^23^ or by inactivation of excess FGF23 in X-linked hypophosphatemic patients,^57–59^ stimulates the mineralization of the non-mineralized bone. Likewise, in the present study, when the LP zebrafish are offered an adequate (RP diet) or increased (HP diet) dietary P, BMD value resumes control value. Histology confirms the resuming of mineralization and the consequent striking increase in mineralized bone volume that exceeds the bone volume increase reported in the previous zebrafish study^24^ (Figure 6B). Interestingly, massive remodeling/resorption is not observed, suggesting that the bone formed is permanent, at least within the time frame of the experiment. Analogous experiments in salmon resulted in the mineralization of previously non-mineralized bone, which yielded vertebral bodies with increased fracture resistance.^23^ Moreover, skeletal malformations are absent both in salmon and in zebrafish. This is an important finding given that the zebrafish vertebral column is subjected to very high levels of mechanical load exerted by muscle contraction during swimming.^60^ Given the spectacular increase in healthy, mineralized bone volume achieved through the alternation of low and sufficient-P intake in zebrafish, it is tempting to test modified conditions on mammalian models to understand how bone elements adapt to the phases of different P intake in relation to gravitational and muscle forces.

Lastly, the LP/sufficient-P dietary intake approach tested here on zebrafish can possibly contribute to design therapeutic approaches aiming to reverse bone loss disorders and bone fragility in humans. However, first studies on murine models are required to fine tune the proper timing for the LP and RP diet phases in mammals. The optimal period of reduced P intake must aim at stimulating sufficient bone formation without inducing rickets/osteomalacia-like side effects. Intermitted optimal periods of reduced dietary P intake could possibly re-establish a healthy bone matrix-to-mineral ratio and to rebuild the osteoid layer that protects bone from resorption, thus resulting in the recovery from bone loss and fragility.

## Supplementary Material

Cotti_et_al_Supplementary_Material_JBMRplus_July24_ziae081

## Data Availability

The data underlying this article are available in the article and in its online supplementary material.

## Notes

Present address: Department of Molecular Medicine, Biochemistry Unit, University of Pavia, Via Taramelli 3b, 27100 Pavia (PV), Italy
