# Supplementary material for: Matrix first, minerals later: fine-tuned dietary phosphate increases bone formation in zebrafish
Source: JBMR Plus. 2024 Jun 18;8(8):ziae081. doi: 10.1093/jbmrpl/ziae081 (PMC11264301; doi:10.1093/jbmrpl/ziae081)
Supplement: Cotti_et_al_Supplementary_Material_JBMRplus_July24_ziae081 [file cotti_et_al_supplementary_material_jbmrplus_july24_ziae081.pdf]

## Supplementary Material

## Supplementary figures

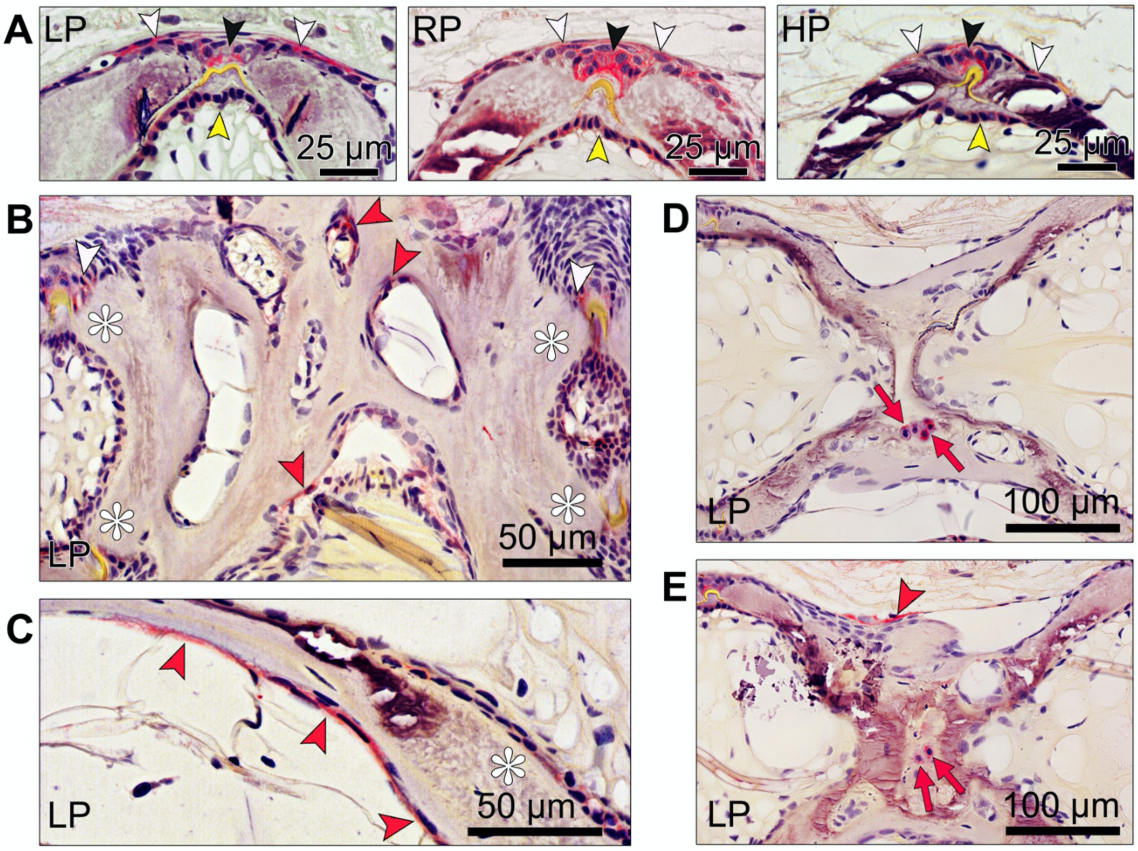


**Fig. S1.** Demonstration of alkaline phosphatase (ALP) activity in the vertebral column of LP, RP and HP zebrafish after two months of dietary treatment. A: In all dietary groups, ALP activity is detected in the vertebral body endplates in specific cell types, i.e., osteoblasts (white arrowheads), fibroblasts (black arrowheads) and cells of the notochord epithelium (yellow arrowheads). B-C: In LP animals, ALP activity (red arrowheads, red staining) is also found in the trabecular bone (B) and on the outer surface of the vertebral centrum (C). Asterisks indicate vertebral body endplates, white arrowheads indicate ALP-positive osteoblasts. D-E: ALP activity is detected in the cartilage-like cells (red arrows) located in LP vertebral bodies that show bent trabeculae.

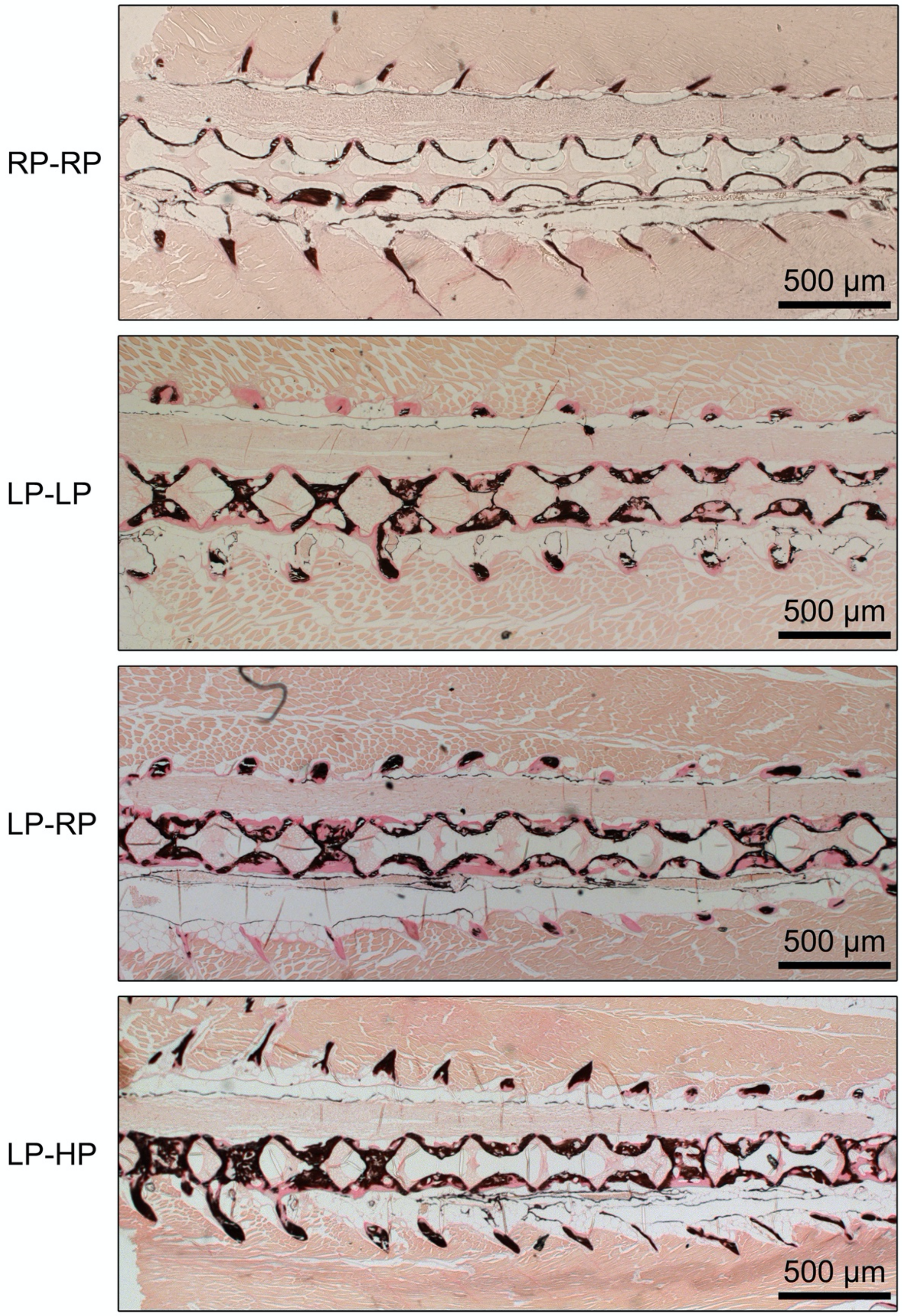


**Figure S2.** Sagittal histological sections stained with Von Kossa/Van Gieson showing the vertebral column of the different dietary groups. Compared to controls (RP-RP), all vertebral bodies in all examined fish with LP dietary history show the extensive bone formation phenotype in a consistent manner throughout the vertebral column. LP-LP zebrafish have more non-mineralized bone matrix compared to the other groups, specially at locations of bone growth (vertebral body endplates and arches). LP-HP show almost a fully mineralized vertebral column, specially at the bone growth zones.

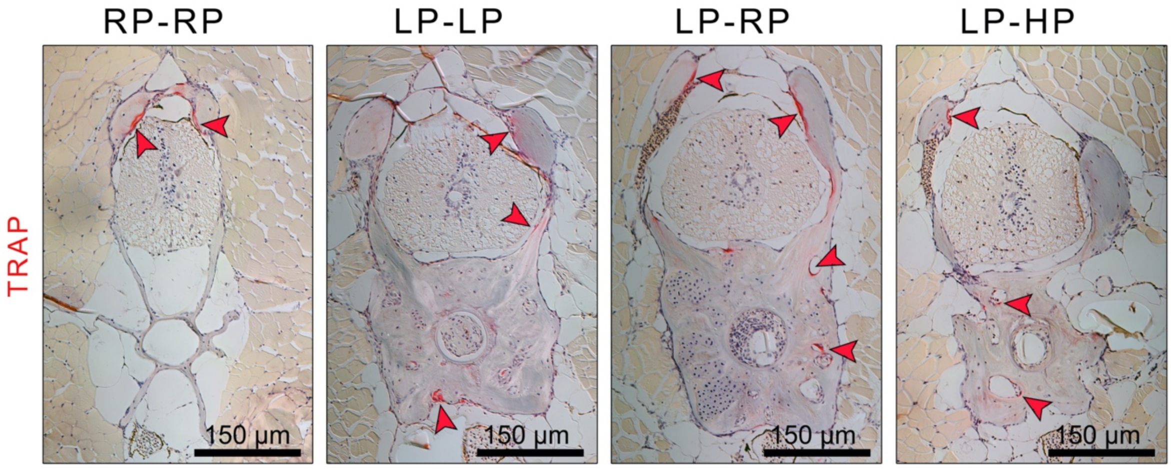


**Figure S3**. Demonstration of tartrate-resistant acid phosphatase (TRAP) activity on sections. TRAP activity (red arrowheads, red staining) is typically detected at endosteal surfaces of enlarging arches, as observed in all dietary groups. In LP-LP, LP-RP and LP-HP zebrafish, TRAP activity is also detected within the bone trabeculae, but increased bone resorption is not observed.

## Supplementary tables

**Table S1.** Primers used for qPCR.

| Gene acronym | NCBI gene accession number | Forward 5'-3' | Reverse 5'-3' |
|---|---|---|---|
| *bglap* | NM_001083857.3 | TGCTGCCTGATGACTGTGTG | GTGCAGTTCCAGCCCTCTTC |
| *col1a1a* | NM_199214.1 | CGTAATGTGCGACGAAGTGA | CTAGGTCCCTCAACACTGGG |
| *sost* | XM_001340647.5 | TACCAGAATACGCGGAGGAC | AAGTCCGTGTGTTTGCTGAC |
| *alpl* | NM_201007.2 | CAGTGGGAATCGTCACAACAA | CCACACAGTGGGCATAAGCA |
| *col10a1a* | NM_001083827.1 | CCGCAGTACCAGCCTTACTC | TTTCCAGGTGCTGAATACCC |
| *sparc* | NM_001001942.1 | CTTCTTCTTGTTCTGCCTCGCT | TCTCAGCAATAACATCCTCCAC |
| *spp1* | NM_001002308.1 | CACAGACAGCGCAGATGACACT | CCCCGGCCTGTGTTGATAATG |
| *ef1a* | NM_131263.1 | TTGAGAAGAAAATCGGTGGTGCTG | GGAACGGTGTGATTGAGGGAAATTC |
| *enpp1* | NM_001030168.1 | TGTGAGCGGACCGATATTTG | TCCATTCAGGACTCCCTTGT |
| *entpd5a* | XM_679770.8 | ATATGCCTGAAAAGGGTGGA | TACTTCTTTGACCTCATTCAGCAG |
| *phex* | NM_001320330.1 | CCGTCATCACGGTATCACAA | TCTGAGCCATGGGTAAATCC |
| *phospho1* | NM_001003461.1 | TGAAAACAGGAGCAGCTGTAAA | GGGGCTGGAGATCTGCTT |

**Table S2.** Bone histomorphometry. Statistical analysis is based on Student's t-test followed by Bonferroni correction; ns: non significant.

| | | Thickness Mean ± SD (µm) | One-way ANOVA | Pairwise p-values | |
|---|---|---|---|---|---|
| Vertebral body endplates | LP | 19.92 ± 2.42 | 0.0011 | LP - RP | 0.0286 |
| | RP | 16.36 ± 1.80 | | LP - HP | 0.0159 |
| | HP | 13.94 ± 0.41 | | RP - HP | ns |
| Vertebral body medial region | LP | 6.27 ± 2.50 | 0.0450 | LP - RP | 0.0571 |
| | RP | 5.00 ± 0.29 | | LP - HP | 0.0286 |
| | HP | 4.23 ± 0.63 | | RP - HP | ns |

**Supplementary Materials and Methods**

*Enzyme histochemistry*

For detection of alkaline phosphatase (ALP) activity on sections, LP, RP and HP fish (n=3 per group) were fixed in 4% PFA in 1× PBS, pH 7.4, for 1 h at RT. Non-decalcified specimens were embedded in glycol methacrylate as described above. Sagittal 5 μm sections were cut on a Microm HM360 (Marshall Scientific, Hampton, New Hampshire, USA) automated microtome and collected on Superfrost Plus slides (Thermo Fisher, Waltam, MA, USA). [(1)] Demonstration of ALP activity was adapted from a published protocol. Briefly, sections were pre-incubated at RT for 1 h in 50 mmol Tris HCl buffer, pH 9.5. The enzymatic reaction took place at RT for 2 h in 50 mmol Tris HCl buffer, pH 9.5, containing 1 mg/mL Naphthol AS-MX phosphate, CAS Number 1596-56-1 (Sigma Aldrich, St. Louis, MO, USA) and 1 mg/mL Fast Red TR salt, CAS Number 51503-28-7 (Sigma Aldrich, St. Louis, MO, USA). Subsequently, slides were rinsed in $dH_2O$, counterstained with Meyers hematoxylin for 10 min, rinsed in running tap water for 10 min, flushed in $dH_2O$, dried at 40°C and mounted with DPX.

**References**

1. Witten PE, Hansen A, Hall BK. Features of mono- and multinucleated bone resorbing cells of the zebrafish *Danio rerio* and their contribution to skeletal development, remodeling, and growth. J. Morphol. 2001;250(3):197–207.
